# The Neural Basis of Individual Face and Object Perception

**DOI:** 10.3389/fnhum.2016.00066

**Published:** 2016-03-01

**Authors:** Rebecca Watson, Elisabeth M. J. Huis in ’t Veld, Beatrice de Gelder

**Affiliations:** Department of Cognitive Neuroscience, Faculty of Psychology and Neuroscience, Maastricht UniversityMaastricht, Netherlands

**Keywords:** face processing, identity recognition, fMRI BOLD, prosopagnosia, categorical perception

## Abstract

We routinely need to process the identity of many faces around us, and how the brain achieves this is still the subject of much research in cognitive neuroscience. To date, insights on face identity processing have come from both healthy and clinical populations. However, in order to directly compare results across and within participant groups, and across different studies, it is crucial that a standard task is utilized which includes different exemplars (for example, non-face stimuli along with faces), is memory-neutral, and taps into identity matching across orientation and across viewpoint change. The goal of this study was to test a previously behaviourally tested face and object identity matching design in a healthy control sample whilst being scanned using fMRI. Specifically, we investigated categorical, orientation, and category-specific orientation effects while participants were focused on identity matching of simultaneously presented exemplar stimuli. Alongside observing category and orientation specific effects in a distributed set of brain regions, we also saw an interaction between stimulus category and orientation in the bilateral fusiform gyrus and bilateral middle occipital gyrus. Generally these clusters showed the pattern of a heightened response to inverted versus upright faces, and to upright, as compared to inverted shoes. These results are discussed in relation to previous studies and to potential future research within prosopagnosic individuals.

## Introduction

It has long been understood that faces are special. From birth we are drawn to faces and recognizing and responding to the information contained within them is something we are particularly good at. For decades now, researchers have attempted to understand the specific cognitive and neural mechanisms underscoring face perception. Combined evidence for the ‘specialness’ of faces has come from a number of different experimental sources, including cognitive psychology, and cognitive and clinical neuroscience.

Cognitive psychology experiments have revealed phenomena such as the part-whole effect, which shows that it is easier to recognize a face part when it is presented as part of a whole face, rather than on its own; the composite effect, which shows that people have difficulties recognizing the top half of a face if it is aligned with a non-corresponding bottom half; and also the face-inversion effect (FIE), where recognition of inverted faces is less accurate than recognition of upright faces. These effects have been used to demonstrate the holistic nature of face processing, which appears to be more marked for faces as compared to other objects (e.g., Yin, 1969). The FIE is of particular interest as compared to the composite and part-whole effects, it appears to tap into a more basic level of configural processing. To explain the effect in more detail, it has been proposed that faces are processed and stored as perceptual wholes, as opposed to a configuration of different individual parts. Thus, when faces are rotated away from an upright orientation holistic processing is impaired, resulting in an inversion effect (e.g., Tanaka and Farah, 1993; Farah et al., 1995a).

Many studies using neuroimaging techniques have highlighted regions with pronounced selectivity for faces brain. Particularly well-documented is an area in the lateral fusiform gyrus where the activity in response to faces is consistently found to be greater than that evoked by non-face objects, which was named the ‘fusiform face area’ (FFA; Kanwisher et al., 1997). Furthermore, some neuroimaging studies have found this region to be affected by orientation manipulations, reporting that the FFA exhibits a greater response to images of upright faces than to images of inverted faces (e.g., Kanwisher et al., 1998; Yovel and Kanwisher, 2005; Passarotti et al., 2007; but also note Aguirre et al., 1999; Haxby et al., 1999; Leube et al., 2003 who did not find such an effect). Although a number of studies have focussed predominately on the FFA, face processing appears to depend on a network consisting of several dedicated cerebral modules, including the occipital face area (OFA), superior temporal sulcus (STS), and face selective areas in the anterior temporal lobe (ATL) and prefrontal cortex (e.g., Kanwisher et al., 1997; Gauthier et al., 2000; Axelrod and Yovel, 2015). An influential model detailed by Haxby et al. (2000) proposed that different regions involved in the face processing network are engaged specifically for different aspects of face processing – for example, face identity processing and face expression processing. This is also supported by electrophysiological research in non-human primates which has highlighted neurons in distinct brain regions that are tuned to expression and orientation, and others identity (Hasselmo et al., 1989; Eifuku et al., 2004).

Furthermore, clinical neuroimaging studies have described patients with selective impairments in the identification of faces. This impairment is referred to as prosopagnosia, a deficit of familiar face recognition. Prosopagnosia can be either acquired – for example, after a brain injury – or developmental, where there is no clear underlying cause; however, in both cases the individuals can perceive a face for a face, but are specifically unable to recognize its identity. In fact, the earliest insights into the neural mechanisms underlying the ability to recognize face identity came from the study of patients with selective impairment for the recognition of faces, with the right occipito-temporal cortex emerging as a common location of the lesion in prosopagnosic patients (e.g., Damasio et al., 1982; Tranel et al., 1997; for a review, see de Gelder and Van den Stock, 2015). It should be noted that support for this region’s involvement in identity processing has also come from functional imaging studies in non-prosopagnosic participants utilizing techniques such as fMR-adaptation (fMR-A; e.g., Grill-Spector et al., 1999; Winston et al., 2004; for a review, see Anzellotti and Caramazza, 2014). A number of studies have now focussed on investigating the FIE in prosopagnosic patients. Specifically, the FIE has been used to assess whether such individuals show impairments in configural perception, and how this is related to feature processing abilities. In theory, one might expect that prosopagnosic patients should show a reduced FIE, or that it should not be present. However, a range of different outcomes have been observed, spanning from a normal effect in one patient, to a reduced/absent effect or even an “inverted” inversion effect in others (see Busigny and Rossion, 2010, for a discussion). In particular, the presence of an inverted inversion effect, or paradoxical inversion effect (specifically, a better performance for inverted faces, e.g., Farah et al., 1995b; de Gelder et al., 1998; de Gelder and Rouw, 2000; Schmalzl et al., 2009) suggests that the prosopagnosic deficit is not simply a loss of configuration perception and its replacement by feature processing, as upright and inverted faces are still being processed differently. Importantly though, Busigny and Rossion (2010) note that discrepancies in results across these studies investigating the FIE may in part be due to differences in patient selection (e.g., using patients with differing general vision ability), tasks utilized, and behavioral measurements that are acquired. Furthermore, to date the neuroimaging evidence regarding the FIE effect in prosopagnosic patients is scarce. This could provide a valuable complement to existing research.

Related to this, it is important to note that when assessing face processing ability in prosopagnosia, several aspects should be taken into consideration. First of all, prosopagnosic individuals are specifically unable to recognize the identity of individual faces. Therefore, presenting a series of single faces and objects, without any focus on identity recognition or matching, merely taps into simple face and object perception. Specifically, when used in combination with fMRI, such a design may inform us about the neural basis of face perception but not about face identity processing. Furthermore, individuals with intact face-recognition skills are usually very well able to distinguish between faces of differing identities, but also non-face stimuli such as cars, houses, shoes, and so forth. What makes these perceptual judgements instances of genuine object recognition is that we can recognize the same object when seen from different viewpoints. Indeed, a hallmark of object recognition is viewpoint invariance, where an object is recognized independently of the viewpoint under which it is presented. To take this into account it is important to prevent “physical identity matching,” which can occur when two identical images are presented together. Experiments designed for prosopagnosic individuals should thus not only present multiple stimuli at the same time, but also from different viewpoints. Furthermore, even if studies do focus on exemplar recognition, they often use one-back or delayed match-to-sample paradigms (Kanwisher et al., 1998; Haxby et al., 1999; Yovel and Kanwisher, 2005; Epstein et al., 2006). Face memory deficits in prosopagnosia have been well documented over the years, but even a short delay in stimulus presentation is disproportionally more detrimental to both the accuracy and reaction times of prosopagnosics as compared to controls (Shah et al., 2015).

In order to study face and object perception with a task suitable for both healthy and prosopagnosic participants, we previously constructed a behavioral face and object perception test battery including an face and object identity matching task, which is a match-to-sample task with upright and inverted faces and shoes (de Gelder and Bertelson, 2009; Huis in’t Veld et al., 2012). In this task, match-to-sample is tested using simultaneously presented sample, target and distractor stimuli, and faces and objects are presented both in the habitual upright and an upside-down orientation, and across varying viewpoints: the sample is seen in frontal view, the target and distractor from a three fourths profile view. This design is optimal as it is memory-neutral, and at the same time uses faces as well as objects, and targets identity matching across orientation and across viewpoint change.

The goal of the current study was to test a version of this face and object identity matching design in a healthy control sample whilst being scanned using fMRI. Here, we were particularly interested to investigate categorical and inversion effects on brain activity; specifically, whether faces and objects have a different neural substrate, whether this is sensitive to orientation, and/or there is neural evidence in one or more areas for a category specific inversion effect, while participants were focused on identity matching of simultaneously presented exemplar stimuli.

## Materials and Methods

### Participants

Sixteen healthy participants (eight males, age range 19–27 years) participated in the study. All had normal or corrected-to-normal vision, and no history of neuropsychiatric disorders. The experiment was approved by the Ethics committee of Maastricht University, and written informed consent was obtained before participation. Participants were screened for fMRI experimentation safety and received monetary compensation for their participants.

### Stimuli and Task

The study consisted of eight experimental conditions with a 2 (category: faces, shoes) by 2 (orientation: upright, inverted) by 2 (congruency: same, different identity) design. The materials consisted of greyscale photographs of shoes (12 unique shoes) taken from the faces and objects matching test (de Gelder and Bertelson, 2009) and faces (six male, six female; all with a neutral facial expression) from a database created at Tilburg University. Each face and each shoe were photographed once in front view and once in three-quarter profile view.

**FIGURE 1:**
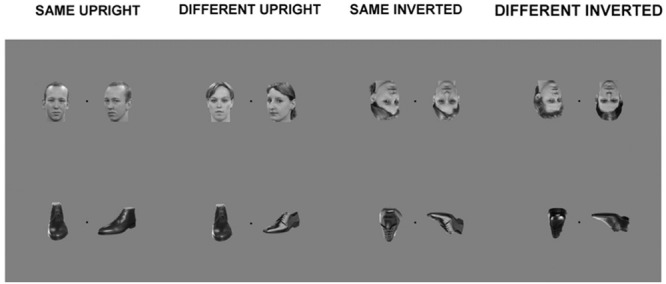
**Examples of stimuli from main experiment.** Each trial within a block consisted of a simultaneous presentation of two within-category stimuli: Matching upright faces; Different upright faces; Matching inverted faces; Different inverted faces; Matching upright shoes; Different upright shoes; Matching inverted shoes; Different inverted shoes.

A trial consisted of one frontal view picture and one three-quarter profile view picture, placed equidistant from a center fixation point, in a counterbalanced way. The span of the two visual images was 16.67 cm by 12.5 cm in total, presented at a visual angle of 12.54°. This presentation was adapted from the behavioral version of the task which consists of a triangular presentation of three stimuli. This adaptation was an attempt to limit excessive eye movement, or saccades of participants, which although cannot be eliminated via simultaneous stimuli presentation, could at least be reduced. Each trial was presented for 800 ms. Participants were asked to fixate at the black center fixation cross between the stimuli and to concentrate on whether the two stimuli were of a matching identity or not, but no response was required (see **Figure 1**, for examples of the stimuli used). Stimuli were presented using Presentation software (Neurobehavioural Systems, San Francisco, CA, USA).

The experimental design was blocked, with four blocks per condition (32 blocks in total) and 12 trials per block. The order of blocks was pseudo randomized; additionally the order of the trials within each block was randomized. Within blocks, the inter-trial interval was 200 ms. Time between blocks was 12000 ms.

### MRI Parameters and Functional Data Processing

MRI was performed using a 3-Tesla Siemens Trio scanner (Siemens, Erlangen, Germany). Both high-resolution anatomical [T1-weighted, flip angle (FA) = 9°, TR = 2250, TE = 2.6 ms, 192 slices, field of view (FoV) = 256 mm, isotropic voxel resolution of 1 mm × 1 mm × 1 mm] and whole-brain functional images [T2*-weighted echo-planar imaging: *TR* = 2000, *TE* = 30 ms, 35 contiguous slices, slice thickness = 3 mm, voxel resolution = 3 mm × 3 mm × 3 mm] were obtained. Participants’ hearing was protected using earplugs, and head movement was restricted using foam pads.

FMRI data were processed using BrainVoyager QX (Brain Innovation, Maastricht, The Netherlands). Pre-processing included slice acquisition time correction, temporal high-pass filtering, rigid-body transformation of data to the first acquired image to correct for motion, and spatial smoothing. Functional data were co-registered to anatomical data per subject, and further transformed to Talairach space.

### Activation Data Analysis

BOLD time courses of 12 s individual blocks were regressed onto a pre-specified model in a conventional GLM. Separate predictors were implemented for four different conditions: Faces-Upright; Faces-Inverted; Shoes-Upright; Shoes-Inverted.

At the group level, we performed a 2 (Category: Face and Shoe) × 2 (Orientation: Upright and Inverted) repeated-measure RFX ANOVA. This analysis allowed us to observe main effects of all three factors, as well as interactions between the conditions. Results were thresholded at *p* < 0.05 FDR corrected for cluster size.

## Results

**FIGURE 2:**
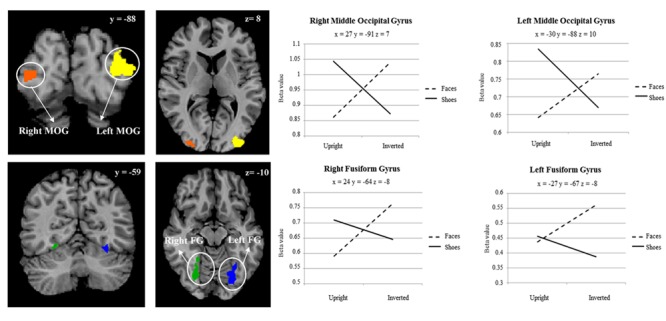
**Two-way interaction between category of stimulus, and stimulus orientation, within a whole brain search.** Plot shows average beta value within cluster for each point measured. Coordinates are in Talairach space.

A main effect of category was found in a wide range of frontal, occipital, temporal, and parietal regions, in addition to the cerebellum, and cingulate and insular cortices. Face specificity was seen in the right fusiform gyrus, right precentral gyrus, right middle frontal gyrus, and right lingual gyrus. For interest, we further investigated these categorical effects using a direct comparison between upright faces and shoes only: here, face specificity was observed in the right inferior frontal gyrus, fusiform gyrus, and left lingual gyrus, whereas shoe (object) specificity was seen in the bilateral fusiform gyrus, extending to the parahippocampal gyrus, and left cuneus. A main effect of orientation was observed in the right postcentral gyrus, middle frontal gyrus, precentral gyrus, claustrum, fusiform gyrus/cerebellum, thalamus, midbrain, posterior cingulate cortex, cuneus, cingulate gyrus and left lentiform nucleus, insula, precentral gyrus, claustrum and precentral gyrus. Generally, all these regions showed a higher response to upright, as opposed to inverted stimuli. We additionally examined inversion effects across category: upright vs. inverted faces elicited activity in the right fusiform gyrus and lingual gyrus, whereas the reverse contrast showed activity in a more posterior region of the right fusiform gyrus. Comparing the response to upright vs. inverted shoes showed higher activation in one cluster in the middle occipital gyrus, whereas the reverse contrast showed no significant activation. Finally, an interaction between both factors emerged in four specific clusters, specifically in the bilateral fusiform gyrus and bilateral middle occipital gyrus. In all of these clusters, the general pattern was that a stronger response was observed for inverted, as opposed to upright faces; and the converse pattern for shoes. All activated clusters are detailed in **Tables 1–3**. Interactions are illustrated in **Figure 2**.

**Table 1:** Results from 2 × 2 repeated measures RFX ANOVA.

Brain region	Direction of Effect	*x*	*y*	*z*	*F*-value	*P*-value	Cluster size
**Main effect of category (faces/shoes)**							
Right (R) precentral gyrus	Face stimuli (F) > shoe stimuli (S)	33	2	31	18.67893	0.000605	2605
R middle frontal gyrus (MFG)	F > S	30	-7	46	20.89781	0.000367	820
R fusiform gyrus (FG)/cerebellum	F > S	36	-49	-17	70.62612	0.00001	2144
R lingual gyrus	F > S	12	-79	-8	42.38495	0.00001	14951
R superior parietal lobule (SPL)	S > F	27	-55	61	22.65684	0.000253	1343
R Cerebellum	S > F	24	-34	-17	65.76097	0.000001	1491
Left (L) medial frontal gyrus (MeFG)	S > F	-3	44	25	47.39478	0.000005	1646
L precuneus	S > F	-6	-46	31	25.28118	0.00015	716
L posterior cingulate cortex (PCC)	S > F	-15	-58	19	27.75431	0.000095	555
L SPL	S > F	-27	-58	58	26.30526	0.000124	2830
L parahippocampal gyrus	S > F	-24	-31	-11	28.79298	0.000079	2632
L angular Gyrus	S > F	-48	-67	28	27.25243	0.000104	1256
L inferior temporal gyrus (ITG)	S > F	-48	-64	-2	23.32776	0.000221	2234
L insula	S > F	-39	-25	-2	21.14508	0.000348	2333
L inferior parietal lobule (IPL)	S > F	-54	-31	40	25.87937	0.000134	1320
**Main effect of orientation (upright/inverted)**							
R postcentral gyrus	Upright orientation (U) > inverted orientation (I)	42	-28	52	18.33478	0.000655	564
R MFG	U > I	42	26	22	18.87688	0.000577	1649
R precentral gyrus	U > I	30	5	28	16.65654	0.000983	682
R claustrum	U > I	36	-7	1	17.02994	0.000896	583
R claustrum	U > I	27	2	16	19.38588	0.000514	511
R FG/cerebellum	U > I	36	-52	-17	40.31436	0.000013	884
R thalamus	U > I	21	-28	-2	18.17756	0.00068	504
R midbrain	U > I	18	-16	-5	14.12844	0.001897	448
R PCC	U > I	18	-55	7	33.70423	0.000035	508
R cuneus	U > I	9	-79	13	15.06105	0.001478	415
R cingulate gyrus	U > I	6	2	43	22.77228	0.000247	794
L lentiform nucleus	U > I	-27	-4	-8	23.01978	0.000235	1390
L precentral gyrus	U > I	-30	-13	37	16.31564	0.00107	421
L insula	U > I	-39	5	16	18.33482	0.000655	751
L precentral gyrus	U > I	-42	-13	28	27.49471	0.000099	551
L claustrum	U > I	-36	-13	1	48.85896	0.000004	535
L precentral gyrus	U > I	-51	-7	13	26.90645	0.00011	522
**Category** ×**orientation interaction**							
R middle occipital gyrus (MOG)	Face inverted > face upright; shoe upright > shoe inverted	27	-91	7	24.19421	0.000185	599
R FG	Face inverted > face upright; shoe upright > shoe inverted	24	-64	-8	30.42105	0.000059	978
L FG	Face upright = shoe upright; face inverted > shoe inverted	-27	-67	-8	25.34552	0.000148	1000
L MOG	Face inverted > face upright; shoe upright > shoe inverted	-30	-88	10	19.07116	0.000552	1587

*Results are thresholded at *p* < 0.05 FDR corrected for cluster size (1000 Montecarlo simulations, input threshold = *p* < 0.01 uncorrected). Coordinates are from the peak voxel of the activated cluster and are reported in Tailarach space. The number of voxels per cluster is reported at the anatomical level.*

**Table 2:** Categorical effects, including only upright stimuli.

Brain region	*x*	*y*	*z*	*T*-value	*P*-value	Cluster size
**Upright faces vs. upright shoes**						
Right (R) inferior frontal gyrus	33	8	25	5.681214	0.000044	736
R fusiform gyrus FG)/cerebellum	36	-49	-17	8.083158	0.000001	1648
L lingual gyrus	-9	-85	-5	6.23081	0.000016	8042
**Upright shoes vs. upright faces**						
R FG^∗^	24	-34	-17	10.4916	0.000000	891
L cuneus	-24	-88	35	4.63019	0.000327	1861
L FG^∗^	-30	-37	-14	5.56953	0.000054	2030

Results are thresholded at *p* < 0.05 FDR corrected for cluster size (1000 Montecarlo simulations, input threshold = *p* < 0.01 uncorrected). Coordinates are from the peak voxel of the activated cluster and are reported in Tailarach space. The number of voxels per cluster is reported at the anatomical level. *Clusters extend to parahippocampal gyrus.

**Table 3:** Orientation effects for each stimulus category.

Brain region	*x*	*y*	*z*	*T*-value	*P*-value	Cluster size
**Upright faces vs. inverted faces**						
Right (R) fusiform gyrus (FG)	36	-46	-14	5.614856	0.000049	778
R lingual gyrus	18	-55	4	7.016321	0.000004	2456
**Inverted faces vs. upright faces**						
R cerebellum/FG	24	-61	-11	-4.67803	0.000297	988
**Upright shoes vs. inverted shoes**						
Left middle occipital gyrus	-30	-94	7	3.780752	0.001813	621
**Inverted shoes vs. upright shoes**						
No significant voxels

*Results are thresholded at *p* < 0.05 FDR corrected for cluster size (1000 Montecarlo simulations, input threshold = *p* < 0.01 uncorrected). Coordinates are from the peak voxel of the activated cluster and are reported in Tailarach space. The number of voxels per cluster is reported at the anatomical level.*

## Discussion

In this study, the purpose was to investigate the neural correlates of face and object perception using a memory-neutral design utilizing both faces and objects of different identities and viewpoints; a design we proposed would be ideally suited for use not only in healthy but also prosopagnosic individuals. Specifically, we aimed to establish the correlates of face-selective effects using this task firstly in participants without any face processing impairments, with the future intention of comparing these results to those with prosopagnosia.

### Category and Orientation Specificity

Regarding category specificity, in a whole brain search we observed a number of distinct regions that responded more to faces as compared to shoes, averaged across orientation: specifically, the fusiform gyrus, precentral gyrus, middle frontal gyrus, and lingual gyrus. Furthermore, when we performed a direct contrast between upright face and shoe stimuli, three clusters with peaks in the inferior frontal gyrus, lingual gyrus, and fusiform gyrus emerged. The observed regions have previously been implicated in the face processing network. For example, face-selective activation in the fusiform gyrus corresponded in location to the well-documented FFA (Kanwisher et al., 1997). Furthermore, the lingual gyrus has been linked to the very early stages of facial processing (Luks and Simpson, 2004), and the precentral gyrus has also been proposed to be part of a large brain network for face recognition (Zhen et al., 2013). Additionally, we found several areas sensitive to the orientation of the stimuli, averaged across category. These included both cortical (e.g., right middle frontal gyrus, bilateral precentral gyrus, right cingulate gyrus, left insula, and right fusiform gyrus) and subcortical (bilateral claustrum, left lentiform nucleus, and thalamus) regions. It should be noted, that interestingly in all of these regions (aside from the right middle frontal gyrus, precentral gyrus, fusiform gyrus, and thalamus) the effect appeared to be driven by a deactivation in response to inverted stimuli, whether these were faces or shoes. However, a different pattern was seen in the category specific inversion effects, as detailed below.

### Category Specific Inversion Effects

In order to explore more the specific effect of stimulus orientation and its relation to stimulus category, we further computed an interaction between these two factors. Four distinct clusters emerged, specifically in the bilateral middle occipital gyrus, and bilateral posterior fusiform gyrus. All these clusters showed the general pattern of responding more to inverted, as opposed to upright faces; additionally, in all of the clusters aside from the left fusiform gyrus there was a contrasting pattern for shoes, in that there was a heightened response to upright as opposed to inverted shoes. The involvement of both purported “face-selective” and “object-selective” cerebral regions in the FIE has been the focus of a number of studies. Some studies demonstrated that the FFA exhibited a greater response to images of upright faces than to images of inverted faces (e.g., Kanwisher et al., 1998; Yovel and Kanwisher, 2005; Passarotti et al., 2007). However, these effects have often been small, and have also not been replicated other studies (e.g., Aguirre et al., 1999; Haxby et al., 1999; Leube et al. 2003), with authors failing to find differences in these two types of stimulus presentation in this region. Interestingly, amongst other clusters we did find in this study that a region within the right fusiform gyrus, appearing to correspond with the FFA, showed a heightened response for upright vs. inverted face stimuli; however, no interaction between category and orientation was observed in this region. A range of studies have now also shown that object recognition and object selective areas [for example, in the lateral occipital complex (LOC)] exhibit greater responses to inverted faces than to upright faces (Aguirre et al., 1999; Haxby et al., 1999; Yovel and Kanwisher, 2005; Epstein et al., 2006). Specifically, Haxby et al. (1999) saw that the only selective effect of face inversion was an increase in activity in extrastriate cortical regions that responded more to houses than to faces. At the same time, effects of face inversion in face-selective regions, and house inversion in house-selective regions, were both small. Similarly, Epstein et al. (2006) found that face inversion lead to greater response in LO and the right middle fusiform object area for inverted versus upright faces but observed no change in the FFA. These results suggest that the presumable additional processing that is required for inverted faces may be undertaken by object-responsive regions. Although we cannot confirm the regions found in our study as ‘face-selective’ or ‘object-selective’ using an independent functional localiser, we would propose the loci of the observed interactions as potentially more object-selective, in line with the studies above.

Single case studies of prosopagnosia following brain damage in adulthood continue to be a very important source of information about the neural basis of face perception. However, findings from different case studies are not always comparable due to differences in fMRI design. To our knowledge, there is not to date a standard task incorporating use of different exemplars of faces as well as of objects, viewpoints, and orientations used in prosopagnosia research. We believe that the present design may help adopting a common platform for such research from where to develop specific hypotheses. Regarding future research, face-selective and inversion effects within object-selective regions are of particular interest for further understanding face-processing in prosopagnosic individuals: for example, these cases could perhaps be expected to show more face-specific activation than controls in object-specific areas, as has been detailed in one study by Dricot et al. (2008) which found activation in an object-specific area when viewing different as opposed to repeated upright faces. In conclusion, we believe that the task detailed in this study provides an interesting starting point for this line of research.

## Author Contributions

BdG designed the experiment, RW collected and analysed data, BdG, RW and EHIV wrote the paper.

## Conflict of Interest Statement

The authors declare that the research was conducted in the absence of any commercial or financial relationships that could be construed as a potential conflict of interest.
